# Wnt3 Is Lipidated at Conserved Cysteine and Serine Residues in Zebrafish Neural Tissue

**DOI:** 10.3389/fcell.2021.671218

**Published:** 2021-05-26

**Authors:** Divya Dhasmana, Sapthaswaran Veerapathiran, Yagmur Azbazdar, Ashwin Venkata Subba Nelanuthala, Cathleen Teh, Gunes Ozhan, Thorsten Wohland

**Affiliations:** ^1^Department of Biological Sciences and Center for BioImaging Sciences, National University of Singapore, Singapore, Singapore; ^2^Izmir Biomedicine and Genome Center (IBG), Dokuz Eylul University Health Campus, Izmir, Turkey; ^3^Izmir International Biomedicine and Genome Institute (IBG-Izmir), Dokuz Eylul University, Izmir, Turkey; ^4^Department of Chemistry, National University of Singapore, Singapore, Singapore

**Keywords:** Wnt3, zebrafish, fluorescence correlation spectroscopy, fluorescence cross-correlation spectroscopy, selective plane illumination microscopy, FCS diffusion law

## Abstract

Wnt proteins are a family of hydrophobic cysteine-rich secreted glycoproteins that regulate a gamut of physiological processes involved in embryonic development and tissue homeostasis. Wnt ligands are post-translationally lipidated in the endoplasmic reticulum (ER), a step essential for its membrane targeting, association with lipid domains, secretion and interaction with receptors. However, at which residue(s) Wnts are lipidated remains an open question. Initially it was proposed that Wnts are lipid-modified at their conserved cysteine and serine residues (C77 and S209 in mWnt3a), and mutations in either residue impedes its secretion and activity. Conversely, some studies suggested that serine is the only lipidated residue in Wnts, and substitution of serine with alanine leads to retention of Wnts in the ER. In this work, we investigate whether in zebrafish neural tissues Wnt3 is lipidated at one or both conserved residues. To this end, we substitute the homologous cysteine and serine residues of zebrafish Wnt3 with alanine (C80A and S212A) and investigate their influence on Wnt3 membrane organization, secretion, interaction and signaling activity. Collectively, our results indicate that Wnt3 is lipid modified at its C80 and S212 residues. Further, we find that lipid addition at either C80 or S212 is sufficient for its secretion and membrane organization, while the lipid modification at S212 is indispensable for receptor interaction and signaling.

## Introduction

Wnts are a class of signaling molecules involved in short- and long-range cell-cell communication that regulates complex tissue patterning during embryogenesis. They coordinate a multitude of developmental processes including cell proliferation, cell polarity induction, lineage specification, cell movement and apoptosis (Clevers and Nusse, 2012; Hikasa and Sokol, 2013). Misregulation of Wnt signaling leads to numerous pathological disorders including cancer (Logan and Nusse, 2004; Azbazdar et al., 2021). To date, 19 wnt genes have been identified in humans and 23 in zebrafish (Miller, 2002; Mikels and Nusse, 2006; Lu et al., 2011). Wnts are ∼ 300-400 amino acids long (molecular weight of ∼40 kDa) with 22-24 conserved cysteine residues that form crucial intramolecular disulfide bonds which are required for their folding and function (Mikels and Nusse, 2006). After translation, Wnts undergo a series of post-translational lipid and sugar modifications in the endoplasmic reticulum (ER) (Kikuchi et al., 2006; Lorenowicz and Korswagen, 2009; Willert and Nusse, 2012; Torres et al., 2019; Wang et al., 2020). Porcupine (Porc), a membrane bound O-acyl transferase (MBOAT), is known to facilitate the addition of lipid moieties to Wnts which confer a hydrophobic nature to Wnts (Tanaka et al., 2000). Oligosaccharyl transferase (OST) appends N-linked glycans to Wnts (Tanaka et al., 2002; Komekado et al., 2007). The post-translationally modified Wnts are subsequently targeted to the cell membrane and secreted. The secreted hydrophobic Wnts navigate the aqueous extracellular matrix and achieve long-range distribution by either binding to HSPG (Mii et al., 2017; Mii et al., 2020), being packaged inside lipoproteins (Panáková et al., 2005), or being shuttled by carrier proteins such as afamins (Mihara et al., 2016), secreted Frizzled Related Proteins (sFRPs) (Üren et al., 2000; Galli et al., 2006) and Secreted Wg-Interacting Molecule (Swim) (Mulligan et al., 2012). Upon reaching their distal target tissues, they are handed over to their cognate receptors and co-receptors. Wnts are known to interact with a wide range of receptors and co-receptors which play a role in deciding the course of Wnt biological activity, of which the Frizzled (Fzd) receptor super-family is the most extensively studied (Niehrs, 2012). Wnts form a complex with the receptor and co-receptor at the cell membrane and activate the downstream signaling cascade for the transcription of genes that regulate embryonic development and tissue homeostasis (Bilić et al., 2007).

A number of studies have reported the role of lipidation in intracellular trafficking, secretion, transport and function of Wnts (Hausmann et al., 2007; Parchure et al., 2018; Hosseini et al., 2019). Wntless (WIs), a functionally conserved transmembrane protein that shuttles Wnts from the Golgi to the cell membrane, is unable to bind to non-acylated Wnt molecules (Bänziger et al., 2006). As a result, non-acylated Wnts are retained in the Wnt producing cells and hence are not secreted (Coombs et al., 2010; Herr and Basler, 2012). Moreover, lipid adducts also help in the long-range distribution and gradient formation of Wnts. For instance, Mulligan et al., 2012 showed that Swim promotes long-range signaling in Drosophila by interacting with Wingless in a palmitate dependent manner (Mulligan et al., 2012). Similarly, evidence suggests that the lipid modifications in Wnt are central to the interaction of Wnts with afamin (Naschberger et al., 2017) and sFRP (Janda and Garcia, 2015). Recently, it was demonstrated how glypicans change conformation to accommodate the lipid tails to facilitate paracrine signaling of Wnts (McGough et al., 2020). In addition to secretion and transport, lipid modifications in Wnts also mediate their interaction with receptors and co-receptors to activate Wnt signaling. The crystal structure of Xenopus Wnt8 (xWnt8) in complex with mouse Fzd8 (mFzd8) revealed that the interaction occurs by xWnt8 projecting its lipid tail into the hydrophobic groove of the mFzd8 cysteine rich domain (CRD) (Janda et al., 2012). The importance of lipid modification on Wnts, from translation to signaling activity, thus initiated detailed investigations on the type and sites of lipid modifications in different Wnts.

Nusse et al. first performed a mass-spectrometric analysis on the proteolytic fragments of purified mouse Wnt3a (mWnt3a) and Drosophila Wnt8 (dWnt8) to identify the type and position of lipidation. They demonstrated that these proteins were modified at their conserved cysteine residue (C77 in mWnt3a and C51 in dWnt8) with a thioester-linked palmitic acid, and mutating this cysteine residue decreased their hydrophobic nature and activity (Willert et al., 2003). Another subsequent mass-spectrometric study by Kurayoshi et al. (2007) further confirmed the addition of palmitate at the homologous C104 residue in mouse Wnt5a. In addition to the palmitoylation at cysteine, Takada et al. reported that mWnt3a is lipidated at a conserved serine residue (S209) with a oxyester-linked palmitoleic acid (Takada et al., 2006). However, a later published 3.25 Å resolution xWnt8-Fzd8 crystal structure identified serine (S187 in xWnt8) as the only lipid addition site, while the corresponding cysteine residue (C55 in xWnt8) was found to be engaged in a conserved disulfide bond making it conformationally unfavorable to serve as a lipid modification site (Janda et al., 2012).

Although the crystal structure recognized serine as the only consensus lipidation site across all Wnts, mutations in the conserved cysteine residue diminishes the hydrophobicity, membrane localization, secretion, and activity in several Wnts. For instance, C93A Wg is retained inside the cells without being secreted and does not activate Wnt signaling in *Drosophila* imaginal disks (Franch-Marro et al., 2008), while C104 mWnt5a and C77A mWnt3a are secreted similar to wildtype but do not interact with Fzd and fail to regulate Wnt pathways in L-cells (Willert et al., 2003; Kurayoshi et al., 2007). MacDonald et al. clarified that a mutation at the conserved cysteine residue results in hydrophilic partitioning and inactive Wnt signaling, due to the aggregation of Wnts by ectopic intermolecular disulfide bonds which buries the lipid adduct in the oligomerized mutant, and not due to the lack of an acyl group at the cysteine residue. However, this does not explain how several Wnt proteins mutated at their homologous serine residues are localized on the cell membrane, are secreted, and remain functional (Franch-Marro et al., 2008; Galli and Burrus, 2011; Tang et al., 2012; Azbazdar et al., 2019; Speer et al., 2019). Given the major structural and functional roles linked with Wnt lipidation, the possibility of an additional lipidation site in at least some Wnt molecules cannot be excluded. To further confound the situation, the impact of inhibiting acylation in Wnts differs with different Wnt ligands and depends on the cellular context. For example, it was recently documented that non-acylated Wnt1 and Wnt5a were unable to initiate Wnt signaling, whereas non-acylated Wnt8 and Wnt3a retained their receptor binding capacity and signaling activity in Xenopus embryos. Furthermore, these results varied when their secretion and activity were examined *in vitro* (Speer et al., 2019). Therefore, it is imperative to characterize lipid modifications for each Wnt ligand in a relevant biological context to comprehend their structure and function.

Wnt3, a member of the Wnt family, is engaged in a number of developmental processes such as primitive streak formation, neurogenesis, vasculogenesis and limb development to name a few (Bulfone et al., 1993; Liu et al., 1999; Garriock et al., 2007; Anne et al., 2013). In zebrafish, Wnt3 is expressed in embryonic neural tissues (Clements et al., 2009; Teh et al., 2015), and our group mapped the expression, dynamics and interactions of zebrafish Wnt3 in vivo to understand its distribution and influence on brain development (Veerapathiran et al., 2020). We had earlier shown in vivo that Wnt3 associates with cholesterol rich domains on the cell membrane and blocking the action of Porc by C59 lowers, but does not abolish, its confinement in the domains in a concentration-dependent manner and results in defective brain development (Teh et al., 2015; Ng et al., 2016). Recently, Azbazdar et al. reported that lipidation of Wnt3 at S212 is dispensable for secretion and binding to Fzd8 receptor in HEK293T cells (Azbazdar et al., 2019). Therefore, it remains unclear whether zebrafish Wnt3 is only lipidated at serine, and how lipidation influences its membrane organization, secretion, receptor binding and activation of Wnt signaling *in vivo*.

To address these questions, we created Wnt3-EGFP constructs mutated at their putative lipidation sites, either cysteine 80 (Wnt3C80A-EGFP) or serine 212 (Wnt3S212A-EGFP) or at both sites (Wnt3C80AS212A-EGFP). These constructs were compared and analyzed for *in vivo* dynamics using confocal fluorescence correlation spectroscopy (FCS), membrane organization using single plane illumination microscopy-FCS (SPIM-FCS), receptor interaction using fluorescence cross-correlation spectroscopy (FCCS) and signaling activity of the lipidation site mutants with Wnt3-EGFP. FCS is a single molecule sensitive technique that measures the concentration and diffusion dynamics of fluorescently labeled molecules in a small observation volume based on the fluorescence fluctuations they create during their transit (Elson and Magde, 1974; Krichevsky and Bonnet, 2002; Enderlein et al., 2004). SPIM-FCS is a multiplexed modality of FCS that records fluctuations from the entire illumination plane in a sample using fast array detectors and generates spatial maps of diffusion coefficients and concentrations in a single measurement which provides information on the membrane organization of a probe (Wohland et al., 2010; Singh et al., 2013; Xue Wen et al., 2017). The ability of the camera-based FCS to bin pixels post-acquisition to create multiple observation areas allows us to easily adapt the FCS diffusion law, a plot of diffusion time against the observation area, to SPIM-FCS (Wawrezinieck et al., 2005; Ng et al., 2015; Veerapathiran and Wohland, 2018). On the other hand, in FCCS, the fluorescence fluctuations of two bound molecules tagged with spectrally different fluorophores are correlated as they transit the observation volume to measure their molecular interaction (Bacia and Schwille, 2007; Ries et al., 2009; Shi et al., 2009; Foo et al., 2012; Schwille and Rigler, 2013). Our results demonstrate that while Wnt3C80A-EGFP and Wnt3S212A-EGFP localize on the cell membrane, albeit with reduced localization to the ordered membrane domains as compared to Wnt3-EGFP, Wnt3C80AS212A-EGFP fails to localize to the cell membrane. While Wnt3C80A-EGFP and Wnt3S212A-EGFP were secreted and were found in the brain ventricle, Wnt3C80AS212A-EGFP was not detected in the extracellular spaces. Finally, we found that Wnt3C80A-EGFP interacts with Fzd1, although with a lower binding affinity as compared to Wnt3EGFP, whereas Wnt3S212A-EGFP did not interact with receptors. However, both mutants fail to activate the Wnt-signaling pathway. Overall, our work suggests that Wnt3 in zebrafish has two lipid modifications at its C80 and S212 residues which play different functional roles. While either residue is sufficient for Wnt3 secretion and membrane localization, the lipidation at S212 seems necessary for receptor interaction and activation.

## Materials and Methods

### Confocal Microscope Setup

An Olympus FV1200 laser scanning confocal microscope (IX83; Olympus, Japan) was used for this study. The samples were illuminated using a 488 nm laser for the EGFP tagged constructs and a 543 nm laser for the mApple tagged constructs. The emitted signal passes through a 120 μm (1 airy unit) pinhole and is filtered by a 510/23 emission filter (Semrock, United States) for EGFP and a 605/55 emission filter (Semrock, United States) for mApple before it reaches the PMT detector for imaging. For FCS and FCCS measurements, emission filters (Semrock, United States) of 510/20 for EGFP and 615DF45 for mApple were used and the emission was detected using a single photon sensitive avalanche photodiode (SPAD; SPCM-AQR-14, PerkinElmer, United States). The signal was recorded and processed using SymPhoTime 64 (PicoQuant, Germany) to calculate the correlation functions. For Quasi PIE-FCCS measurements, the sample was illuminated simultaneously using a 485 nm pulsed laser (LDH-D-C-488; PicoQuant) at 20 MHz repetition rate and the 543 nm continuous wave laser of the confocal microscope. The emission was separated using a dichroic mirror 560 DCLP and was directed towards a 510/23 (Semrock, United States) or a 615DF45 filter (Semrock, United States) for the different detection channels. The auto- and cross-correlation functions were calculated using a custom written program in Igor Pro (Wavemetrics, United States^1^.

### Confocal Fluorescence Correlation Spectroscopy

Fluorescence correlation spectroscopy extracts information such as the concentration and dynamics of fluorescent molecules from the fluorescence intensity fluctuations in a small observation volume. The fluctuations are generated from processes such as translational diffusion, rotational diffusion, flow, chemical reactions, and fluorophore blinking. The intensity fluctuations are temporally autocorrelated to extract the information embedded in them. The autocorrelation function (ACF) is given by

(1) $$G\,\left(\tau \right)=\frac{\left\langle F\,\left(t\right)\cdot F\,\left(t+\tau \right)\right\rangle }{\left\langle F\,\left(t\right)\right\rangle \cdot \left\langle F\,\left(t+\tau \right)\right\rangle }$$

where F(t) is the intensity at time t and τ is the lag time. The actual form of the ACF can be derived for different processes generating the fluctuations. For a 3D free diffusion of a single species (3D – 1 particle – 1 triplet model) with a triplet state, the theoretical ACF is given by

(2) $$G\,{\left(\tau \right)}_{3\,D,1\,p,1\,t}=\frac{1}{N}\,{\left(1+\frac{\tau }{\tau_{d}}\right)}^{-1}\,{\left(1+\frac{\tau }{K^{2}\,\tau_{d}}\right)}^{-\frac{1}{2}}\,f_{t\,r\,i\,p}\,\left(\tau \right)+G_{\infty },$$

where N is the number of molecules in the observation volume, τ_*d*_ is the diffusion time of the molecule, *G*_*∞*_ is the convergence at long lag times, K is the structure factor which determines the shape of the confocal volume and f_trip_ (τ) is the triplet state function given by

(3) $$f_{\text{trip}}\,\left(\tau \right)=1+\frac{F_{\text{trip}}}{1-F_{\text{trip}}}\,e^{-\frac{\tau }{\tau_{\text{trip}}}}.$$

where, F_trip_ is the fraction of particles in the triplet state and τ_trip_ is the triplet relaxation time.

In case of two diffusion components, the correlation function for a two-component 3D diffusion process is

(4) $$G{\left(\tau \right)}_{3\,D,2\,p,1\,t}=\frac{1}{N}[\left(1-F_{2}\right){\left(1+\frac{\tau }{\tau_{d\,1}}\right)}^{-1}{\left(1+\frac{\tau }{K^{2}\,\tau_{d\,1}}\right)}^{-\frac{1}{2}}+F_{2}{\left(1+\frac{\tau }{\tau_{d\,2}}\right)}^{-1}{\left(1+\frac{\tau }{K^{2}\,\tau_{d\,2}}\right)}^{-\frac{1}{2}}]f{}_{\text{trip}}(\tau )+G{}_{\infty }$$

where F_2_ is the fraction of the second component (3D – 2 particle – 1 triplet model). For two-dimensional diffusion, such as on the cell membrane, equations 2 and 4 become

(5) $$G\,{\left(\tau \right)}_{2\,D,1\,p,1\,t}=\frac{1}{N}\,{\left(1+\frac{\tau }{\tau_{d\,1}}\right)}^{-1}\times f_{\text{trip}}\,\left(\tau \right)+G_{\infty }$$

and

(6) $$G{\left(\tau \right)}_{2\,D,2\,p,1\,t}=\frac{1}{N}[\left(1-F_{2}\right){\left(1+\frac{\tau }{\tau_{d\,1}}\right)}^{-1}+F_{2}{\left(1+\frac{\tau }{\tau_{d\,2}}\right)}^{-1}]f{}_{\text{trip}}+G{}_{\infty }$$

and are referred to as 2D – 1 particle – 1 triplet model and 2D – 2 particle – 1 triplet model, respectively. The system was first calibrated with 5 nM Atto 488 dye (ATTO-TEC GmbH, Germany) for the 488 and 485 nm lasers and 5 nM Atto 565 dye (ATTO-TEC GmbH, Germany) for the 543 nm laser line. The ACF for the calibration dyes (D of 400 μm^2^/s) was fit using equation 2 and the free fit parameters were N, τ_*d*_, τ_trip_, F_trip_, and G_∞_. All measurements were taken at room temperature with an acquisition time for each measurement of 60 s. For FCS measurements, the fitting models were chosen by Bayes inference-based model selection which determines the most suitable model given the data and noise (Sun et al., 2015). The fit models selected for Wnt3S212A-EGFP, Wnt3C80A-EGFP and Wnt3-EGFP was a 2D – 2 particle – 1 triplet model for measurements in cell membranes, while a 3D – 2 particle – 1 triplet model was used for measurements in the BV. For secEGFP measurements, a 3D – 1 particle – 1 triplet model was used. The laser power used for all FCS measurements was 8.3 μW of 488 nm continuous wave laser.

### Quasi PIE Fluorescence Cross-Correlation Spectroscopy

Fluorescence cross-correlation spectroscopy is useful in studying the interaction between molecules in live samples. When two interacting molecules tagged with spectrally different fluorophores pass through the confocal volume, the fluorescence intensity fluctuations from the two channels can be cross-correlated to obtain the cross-correlation function (CCF), G_*x*_ (τ), given by

(7) $$G_{x}\,\left(\tau \right)=\frac{\left\langle F_{g}\,\left(t\right)\cdot F_{r}\,\left(t+\tau \right)\right\rangle }{\left\langle F_{g}\,\left(t\right)\right\rangle \cdot \left\langle F_{r}\,\left(t\right)\right\rangle }$$

where *F*_*g*_ and *F*_*r*_ represents the fluorescence intensity in the green and red channels, respectively. The ACFs were fitted using 2D-2particle-1triplet model as described in equation 6.

In order to determine interaction of Wnt3S212A-EGFP and Wnt3C80A-EGFP with Fzd1, a pulsed 485 nm laser line and a continuous 543 nm laser line were simultaneously used to excite EGFP and mApple, respectively. The intensity in each channel were autocorrelated to obtain their respective ACF and cross-correlated to obtain the CCF. Fluorescence lifetime correlation spectroscopy (FLCS) based statistical filtering was applied to eliminate spectral cross talk (Padilla-Parra et al., 2011; Kapusta et al., 2012). The apparent dissociation constant (K_*d*_) was calculated as detailed in Veerapathiran et al. (2020). Laser power used for FCCS measurements were 8.8 μW for 485 nm pulsed laser and 8.5 μW for 543nm continuous wave laser.

### Sample Preparation for Diffusion Law Analysis on SPIM

Early-stage zebrafish embryos expressing Wnt3S212A-EGFP, Wnt3C80A-EGFP and Wnt3-EGFP were taken at 2-3 days-post-fertilization (dpf) and anesthetized in MS-222 (Merck) solution. These embryos were placed in low melting 1% agarose (UltraPureTM Low Melting Point Agarose, 16520100, Thermofisher Scientific, United States) mixed with MS-222 solution and mounted into a Fluorinated Ethylene Propylene (FEP) tube of 1.1 × 1.5 mm^2^ cross-section (FT 1.1 × 1.5, Adtech Polymer Engineering, England, United Kingdom) with the help of a plastic wire in such a way that their dorsal hind brain region faced one of the walls of the FEP tube. In such a position, the light sheet illuminated the coronal plane of the embryo and the detection objective faced and thus imaged the dorsal region of the embryo.

### SPIM Setup

A home-built SPIM described in Singh et al. (2013) and Krieger et al. (2015) was used to measure the membrane organization and dynamics of Wnt3 and its lipidation site mutants. The system contains an illumination arm that creates a Gaussian light sheet to illuminate the sample in the sample chamber, and a detection arm with the detection objective, tube lens and a camera to image the embryo. A 488 nm diode laser line (Cobolt 06-MLD 488nm 0488-06-01-0100-100, Cobolt AB, Sweden) was used for all measurements. This was directed through an optical fiber (kineFLEXP-3-S-405..640-1.0-4.0-P2, Qioptiq, United States). The beam from this fiber was reflected onto the illumination arm with a 45-degree mirror and passed through an achromatic cylindrical lens of 75 mm focal length (ACY254-075-A; Thorlabs Inc., United States) and an illumination objective (SLMPLN 20 × /0.25; Olympus, Japan) to form the light sheet. The light sheet thickness obtained had a 1/e^2^ radius of approximately 1.1 μm. The thinnest section of the light sheet was aligned to match the focal plane of the detection objective (LUMPLFLN 60 × /1.0, Olympus, Japan).

The embryo was mounted in the FEP tube that was held by a self-closing forceps and was mounted on a motorized stage with three linear positioning systems (Q-545 Q-MotionR Precision Linear Stage; Physik Instruments, Germany) with piezo motors for the three axis and one rotation stage (DT-34 Miniature Rotation Stage; Physik Instruments, Germany). The sample chamber was a 3 cm × 3 cm × 3 cm cube with an opening on top for mounting the sample and an opening on the side for the detection objective. The sample chamber was filled with water and the embryos were lowered into the chamber from the top with the motorized stage. The detection objective was placed in a mounting hole on one side of a custom-made sample chamber and mounted on a piezo flexure objective scanner (P-721 PIFOC; Physik Instruments, Germany) for controlling the position of the detection objective with respect to the light sheet. The emission obtained by the detection objective was passed through a filter (FF03-525/50-25, Semrock, United States) and was projected onto an EMCCD camera (Andor iXon3 860, 128 x 128 pixels; Andor Technology, United Kingdom) by a tube lens (LU074700, *f* = 180 mm, Olympus, Tokyo, Japan). A flip mirror was used to alternatively project the image onto a sCMOS camera (OCRA-Flash4.0 V2; C11440, Japan) with a large field of view to visualize the global position of the embryo and to decide on the region of interest.

### Data Acquisition and Fitting for SPIM-FCS

**FIGURE 1:**
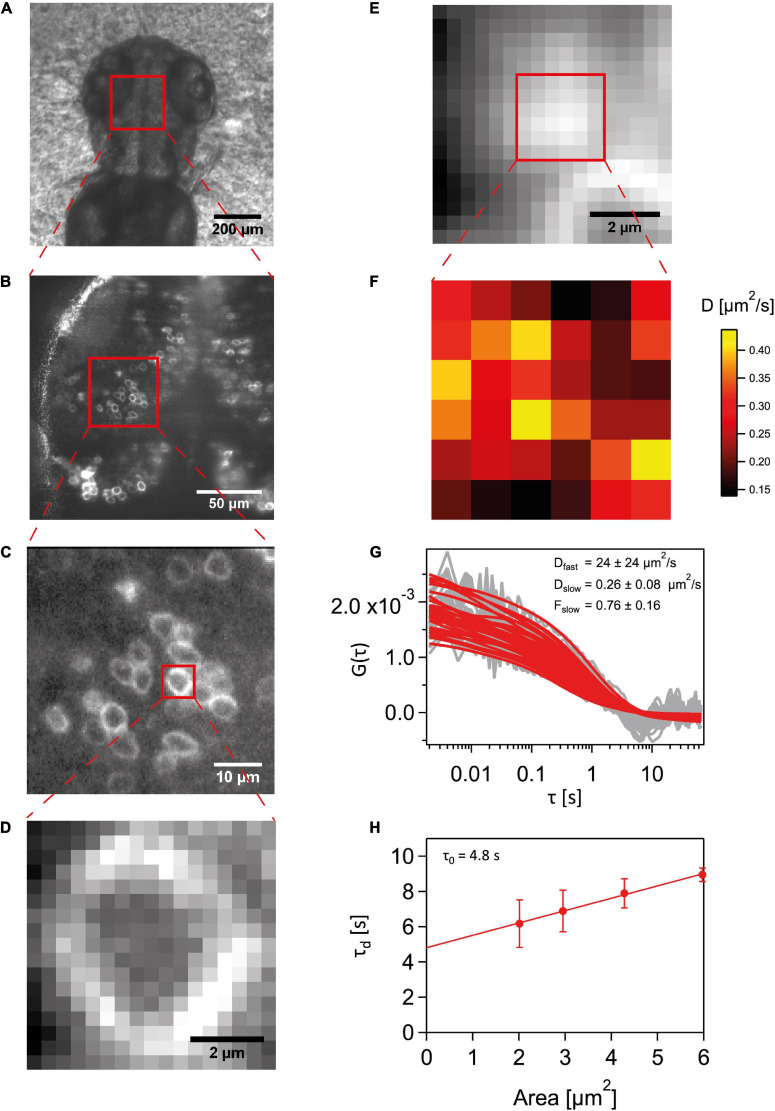
Schematic for a SPIM-FCS experiment. **(A)** A stereomicroscope image of the dorsal region of a 2-3 dpf Wnt3-EGFP expressing embryo. **(B)** sCMOS camera image capturing a large field of view of Wnt3-EGFP expressing cells at the midbrain-anterior hind brain region. **(C)** EMCCD camera image of a smaller subregion expressing Wnt3-EGFP fluorescent cells. **(D)** Cropped detector region of the imaging area with only a single cell in view. **(E)** A summed-up intensity projection of the 100,000 frames imaging the apical membrane of the cell imaged in **(D)**. **(F)** A 6 6 subregion’s slow component diffusion coefficient map obtained from the fit values of the ACFs. **(G)** The raw autocorrelation data in gray and their corresponding fits in red. **(H)** Diffusion law plot for the 6 6 region to obtain the τ_0_ intercept value.

An illustration for a general sequence of steps involved in SPIM-FCS and diffusion law measurements is given in Figure 1. Fluorescent cells in the cerebellum and midbrain of 2–3 dpf embryos were chosen to perform SPIM-FCS measurements. The embryo samples experienced a laser intensity of ∼60 W/cm^2^, which was used on the majority of the samples except for nine cells expressing Wnt3S212A-EGFP wherein intensity of 120 W/cm^2^ was used. The global region of interest was identified by using a sCMOS camera allowing for a large field of view. Cells expressing the fluorescent molecules of interest were chosen and then imaged on the EMCCD camera to perform Imaging FCS to take advantage of the signal amplification from the EM gain. The acquisition field was cropped to image only the cell of interest and the sample mounting stage was moved in steps of 300 nm to image a region on the apical membrane. 100,000 frames were recorded with an exposure time of 2 ms. For analysis, the image stacks were summed up to obtain an intensity projection to find the region containing only the membrane and then the image stacks were accordingly cropped to 6 × 6 pixels. The intensity traces of each pixel were corrected for bleaching and were autocorrelated using the ImageJ plugin Imaging FCS 1.52^2^. Data from cells drifting during the measurement or having significant traces of mechanical vibrations in their autocorrelations were excluded.

These ACFs were fit with a 2-component fitting model. Here, the fast component was captured by a 3D free diffusion fitting model to account for the dynamics of molecules in the intracellular and extracellular space. Diffusion on the membrane was accounted for by using a 2D free diffusion fitting model for the slow component (Sankaran et al., 2009). The fitting function is given below.

(8) $$G{\left(\tau \right)}_{S\,P\,I\,M}=\frac{1}{N}[\left(1-f_{2}\right)p\left(D_{1},\tau \right){\left(1+\frac{4\,D_{1}\,\tau }{\omega_{z}^{2}}\right)}^{-\frac{1}{2}}+f_{2}p\left(D_{2},\tau \right)]+G{}_{\infty }$$

where

(9) $$p\left(D,\tau \right)=[\frac{\sqrt{4\,D\,\tau +\omega_{x\,y}^{2}}}{a\,\sqrt{\pi }}\left(e^{-{\left(\frac{a}{\sqrt{4\,D\,\tau +\omega_{x\,y}^{2}}}\right)}^{2}}-1\right)+erf\left(\frac{a}{\sqrt{4\,D\,\tau +\omega_{x\,y}^{2}}}\right)]{}^{2}$$

Here, N is a fitting parameter that represents the total number of particles in the observation volume, D_1_ and D_2_ are the 3D fast component and the 2D slow component’s diffusion coefficient fitting parameters respectively, f_2_ is the fraction of the slow component fitting parameter and *G*_*∞*_ is the fitting parameter for obtaining the convergence value at infinite lag times. ω_*xy*_ is the PSF of the objective (644 nm) and ω_*z*_ is the 1/e^2^ thickness of the light sheet (∼1.1 μm). The pixel size of the EMCCD was 24 μm and with a magnification of 60 × each pixel effectively imaged 0.4 μm in the sample (represented as ‘a’ in equation 9). As the acquisition time of 2 ms was much too slow to detect triplet fluctuations, that component of dynamics was not included in the fitting function unlike the confocal FCS fitting model.

The bleach correction model typically used to linearize a decaying trace is a polynomial function. However, the polynomial function cannot correct for infrequently observed clusters which cause transient intensity bursts. These clusters change the shape of the ACF curve and results in an apparent low diffusion coefficient. As we observed clusters in our samples, we used a line segment-based bleach correction that divides the intensity trace into equal segments, linearizes each segment and concatenates the linearized intensity trace. This mostly abolishes the influence of the aggregates on the correlation functions. We chose a line segment size of 5000 data points as this removes the influence of the rare clusters and other slow fluctuations but leaves the fast and slow diffusion coefficients unchanged. If line segments much smaller are chosen, the line segment correction overcorrects resulting in diffusion coefficient overestimations.

### SPIM-FCS Diffusion Law

The theory of the FCS diffusion law was initially developed by Lenne and colleagues (Lenne et al., 2006) and later adapted for camera-based FCS modalities by our group (Ng et al., 2015, 2016; Bag et al., 2016; Veerapathiran and Wohland, 2018). Briefly, it characterizes a molecule’s mode of diffusion as free diffusion or transiently trapped domain confined diffusion or cytoskeleton meshwork hindered hop-diffusion based on the dependence of the probe’s diffusion coefficient. The FCS diffusion law is a plot of diffusion time (τ_d_) versus effective observation area (A_*eff*_) (equation 10). In camera-based FCS, A_*eff*_ is the convolution of the PSF with the detection area, and a wide range of A_eff_ is obtained from a single FCS measurement by software binning of the pixels after image acquisition. The τ_d_ for each observation area is plotted and by analyzing the *y*-intercept value (τ_0_) we can classify the mode of diffusion as free diffusion (τ_0_ = 0), domain confined diffusion (τ_0_ > 0) or cytoskeleton influenced hop-diffusion (τ_0_ < 0).

(10) $$\tau \,\left(A_{\text{eff}}\right)=\tau_{0}+\frac{A_{\text{eff}}}{D_{\text{eff}}}$$

Larger bin sizes have sufficient area to capture the fluorescence fluctuations coming from the movement of molecules in and out of an observation volume. As 1x1 region exhibited larger variability, the diffusion coefficient from that point was not included for the diffusion law. Pixel binning from 2 × 2 to 5 × 5 was used instead to obtain different A_eff_ and their corresponding τ_d_ values. The slower diffusion component pertaining to that of the membrane was used as D_eff_ to obtain the τ_d_ values which were then plotted against A_eff_ values. The plots were fit to a straight line to obtain the τ_0_ intercept value. Diffusion law measurements with negative slopes or that resulted in highly nonlinear convex or concave curves resulted in either extremely high or low diffusion law intercepts. This happened in less than 25% of all measurements and these were excluded as they were judged to result from membrane curvature and thus a non-flat geometry of the membrane within the light sheet.

### Generation of Transgenic Lines and Constructs

A stable wnt3 promoter-driven line was generated as described (Teh et al., 2015) by PCR amplification of Wnt3-EGFP and pEGFPN2 fusion with HindIII and KpnI flanked primers: HindIII_Wnt3F, 5′-aagcttATGGATTTGTACCTGGTTGGAT-3′ and KpnI_Wnt3R, 5′ggtaccaaTTTACATGTATGTACGTCGTAGA3′. From this, Wnt3-EGFP SV40 polyA fragment was excised to prepare the 4-kbWnt3EGFP-miniTol2 recombinant plasmid.

SecEGFP sequence was also obtained in a similar way by replacing Wnt3EGFP ORF with secEGFP into the 4-kbWnt3EGFP-miniTol2 recombinant plasmid which resulted in a 4-kbsecEGFP-miniTol2 recombinant plasmid which was co-injected with Tol2 transposase at 1–2 cell stage for 4 kb wnt3 promoter-driven somatic expression of SecEGFP.

The constructs for the mutants (Wnt3C80A-EGFP and Wnt3S212A-EGFP) were created using by means of site directed mutagenesis targeting two base pairs of TGT (cytosine) into GCT (alanine) of wnt3 for Wnt3C80A-EGFP and targeting one base pair of **T**CA (serine) into **G**CA (alanine) of Wnt3 gene of Wnt3S212A-EGFP in the recombinant DNA vector (pminiTol2 containing 4 kb upstream zebrafish Wnt3 promoter driving the expression of Wnt3-EGFP).

Wnt3S212A-EGFP was generated by site directed mutagenesis at wt Wnt3-EGFP by targeting 2 basepairs, TGT (cytosine) into **GC**T (alanine), of Wnt3 gene. The following primers were used: forward: 5′CCATGGGCTGgCAGGCAGCTG3′ and reverse: 5′CATTTACAGCGCAGGTGCATGTTC3′. The PCR product was ligated into pGFP-N2-zfWnt3-S212A vector. Wnt3C80A-EGFP was generated by side directed mutagenesis at wt Wnt3-EGFP by targeting 1 basepair of **T**CA (Serine) into **G**CA (alanine) of Wnt3 gene of Wnt3S212A-EGFP in the recombinant DNA vector (pminiTol2 containing 4 kb upstream zebrafish Wnt3 promoter driving the expression of Wnt3-EGFP). The following primers were used: forward: 5′TATCCAGGAGgcTCAGCACCAGTTCC3′ and reverse: CCCAGTTTGACTCCTTCC. The PCR product was ligated into pGFP-N2-zfWnt3-C80A.

For Wnt3C80AS212A-EGFP, mutated gene of both C80A and S212A were amplified as inserts using following primers from pGFP-N2-zfWnt3-C80A and pGFP-N2-zfWnt3-S212A, respectively: Insert-C80A(mTol2) forward primer: ATGGATTTGTACCTGGTTGG and Insert-C80A (mTol2) reverse primer: GCAAGAAAGAAAACTAGAGATTC TTGTTTgcggccgctctagagtcgcggccgctttact. Pminitol2-4kbwn t3pro plasmid was amplified with respective primers for both C80A and S212 A separately using following primers: Vector_tol2_reverse primer: TCCAACCAGGTACAAATCCA TggtggcgaccggtggatcG Vector_Tol2_forward primer: agcg gccgcAAACAAGAATCT using Gibson assembly.

### Microinjection and Zebrafish Sample Preparation

All wild type zebrafish (*Danio rerio*) were maintained at 28°C and embryos were obtained by allowing them to mate naturally according to the University’s animal care protocol (BR18-1023). 40 ng of required plasmid was injected into the zebrafish embryos at one-cell stage and the embryos were incubated at 28°C. All the embryos were screened for fluorescence before measurement at 48pf and embryos older than 24 hpf were treated with 1-phenyl-2-thiourea to prevent melanin formation and subsequent pigmentation. Healthy embryos, dechorionated and anesthetized with MS-222 (Merck), were laterally mounted on a glass bottom dishes (MATEK dish) in 1% low gelling agarose (Merck).

### Capped Sense mRNA Synthesis and Whole-Mount *in situ* Hybridization (WMISH)

Capped sense RNAs of wt Wnt3-EGFP, Wnt3C80A-EGFP and Wnt3S212A-EGFP were synthesized with mMessage mMachine Kit (Thermo Fisher Scientific, Waltham, MA, United States). For phenotypic observation and WMISH, 100 pg of mRNA was injected into one-cell transgenic Tg(7xTcf-Xla.Siam:nlsm-Cherry^*ia*^) zebrafish embryos (Moro et al., 2012). The Wnt antagonist IWR-1 was added to the embryo water at 8 h-post-fertilization (hpf) and kept until 24 hpf. Embryos were initially photographed at 24 hpf under a Zeiss Stemi 508 stereo microscope (Carl Zeiss AG, Jena, Germany). Next, embryos were fixed at 24 hpf in 4% paraformaldehyde (PFA) dissolved in PBS overnight. WMISH was performed with mCherry antisense RNA probe as described previously (Jowett and Lettice, 1994).

## Results

### C80A and S212A Mutations Do Not Affect Wnt3 Secretion *in vivo*

**FIGURE 2:**
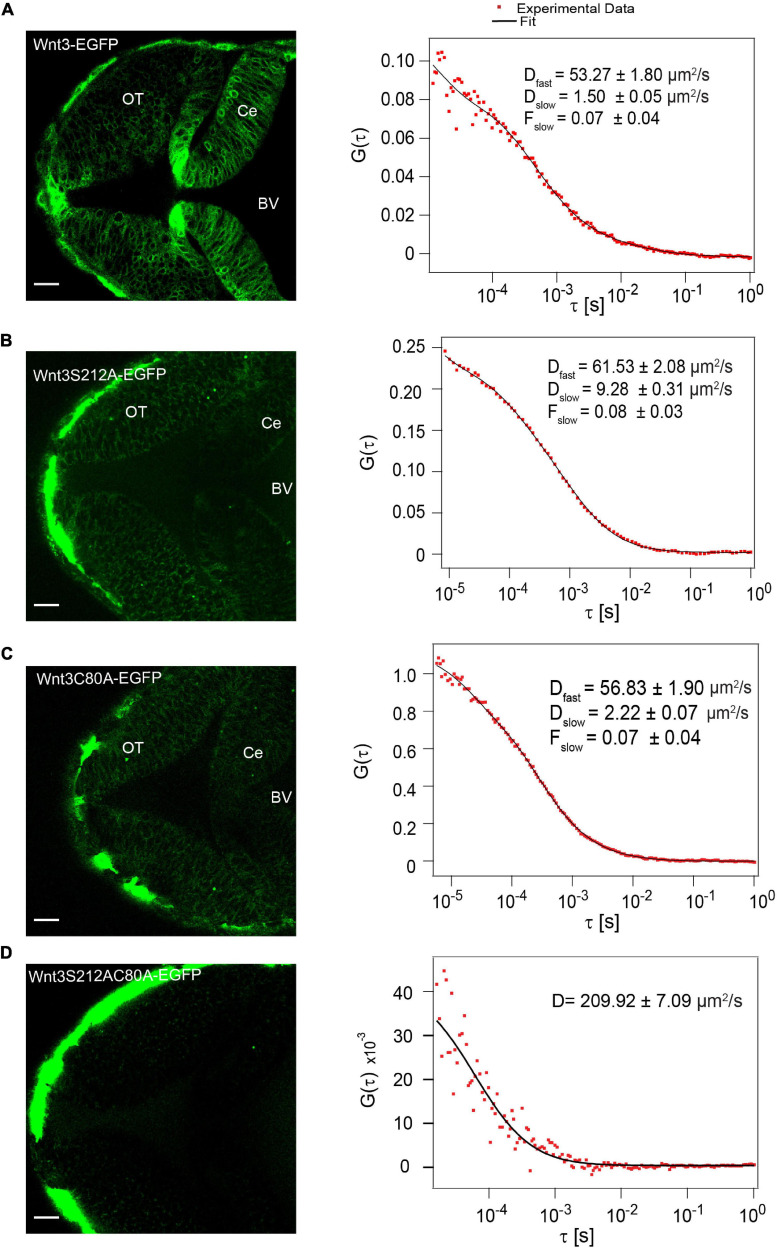
Influence of C80 and S212 lipidation on secretion *in vivo*. **(A)** Expression of Wnt3-EGFP in the zebrafish brain at ∼48 hpf (left) and a representative autocorrelation function (ACF; dots) and fit (line) of a Wnt3-EGFP FCS measurement in the BV (right). **(B)** Expression of Wnt3S212A-EGFP in the zebrafish brain at ∼48 hpf (left) and a representative autocorrelation function (ACF; dots) and fit (line) of Wnt3S212A-EGFP FCS measurement in BV (right). **(C)** Expression of Wnt3C80A-EGFP in the zebrafish brain at ∼48 hpf (left) and a representative autocorrelation function (ACF; dots) and fit (line) of a Wnt3C80A-EGFP FCS measurement in the BV (right). **(D)** Expression of Wnt3S212AC80A-EGFP in the zebrafish brain at ∼48 hpf (left) and a representative autocorrelation function (ACF; dots) and fit (line) of Wnt3S212AC80A-EGFP FCS measurement in BV (right). The FCS curves were fitted using 3D-2particle-1triplet model. BV, fourth brain ventricle; Ce, cerebellum; OT, optic tectum. Orientation: anterior to the left. Scale bar 20 μm.

To study the influence of lipidation on the action of zebrafish Wnt3 *in vivo*, we first examined whether a single point mutation at the putative lipidation sites perturbs Wnt3 secretion *in vivo*. We injected one-cell stage wildtype (WT) embryos with either Wnt3C80A-EGFP or Wnt3S212A-EGFP plasmids and determined via confocal FCS if they are secreted in the fourth brain ventricle (BV). The BVs are cerebrospinal fluid filled cavities enriched with several extracellularly secreted signaling factors, including Wnt (Ochoa et al., 2015; Kaiser et al., 2019; Kaiser and Bryja, 2020). We had earlier demonstrated that functional Wnt3-EGFP, driven by the 4 kb wnt3 promoter in the transgenic line Tg (-4.0wnt3:Wnt3EGFP), is secreted extracellularly and diffuses in the BV (Teh et al., 2015; Veerapathiran et al., 2020) as two fractions: a fast component with a diffusion coefficient D_fast_ of 56.9 ± 11.9 μm^2^/s and a slow fraction with a D_slow_ of 3.6 ± 2.6 μm^2^/s (fraction of slow component F_slow_ = 0.1 ± 0.1) (Teh et al., 2015). Therefore, we performed FCS in the BV to detect if the mutants are secreted similar to Wnt3-EGFP. We obtained autocorrelation functions (ACF) for both Wnt3C80A-EGFP and Wnt3S212A-EGFP, suggesting that the mutants are secreted into the BV (Figure 2). The correlation functions for the mutant constructs were fitted with a 3D-2particle-1triplet model (see Materials and Methods), and the D for both components were computed (see Table 1). We observed a fast diffusing component (D_fast_ of 52.5 ± 10.3 μm^2^/s for Wnt3C80A-EGFP; 54.4 ± 10.4 μm^2^/s for Wnt3S212A-EGFP) and a slow diffusing component (D_slow_ = 6.8 ± 6 μm^2^/s and F_slow_ = 0.3 ± 0.2 for Wnt3C80A-EGFP; D_slow_ = 4.6 ± 2.0 μm^2^/s and F_slow_ = 0.3 ± 0.1 for Wnt3S212A-EGFP). However, when we injected the double mutant Wnt3C80AS212A-EGFP construct at the one-cell stage and performed FCS measurements in the BV, we observed correlation functions characteristic for autofluorescence with a very fast diffusion coefficient (∼200 μm^2^/s in Wnt3C80AS212A-EGFP injected embryos, compared to 225 ± 56.1 μm^2^/s for wild type un-injected embryos) that are unphysical for proteins of a size of Wnt3 (Figure 2D).

**TABLE 1:** Diffusion coefficients of Wnt3-EGFP, Wnt3C80A-EGFP and Wnt3S212A-EGFP in cell membrane and fourth brain ventricle as measure by confocal FCS.

Region	Sample	D_fast_	D_slow_	F_slow_	No. of measurements
Cell Membrane	Wnt3-EGFP	31 ± 9	0.60 ± 0.2	0.60 ± 0.05	14 (3)
	Wnt3S212A-EGFP	28 ± 5	0.7 ± 0.3	0.50 ± 0.06	17 (4)
	Wnt3C80A-EGFP	27 ± 8	0.6 ± 0.2	0.60 ± 0.04	24 (4)
Brain Ventricle	Wnt3-EGFP	57 ± 12	3.6 ± 2.6	0.1 ± 0.1	16 (3)
	Wnt3S212A-EGFP	54 ± 10	4.6 ± 2.0	0.3 ± 0.1	13 (4)
	Wnt3C80A-EGFP	53 ± 10	6.8 ± 6.0	0.3 ± 0.2	11 (4)

*Data are mean ± S.D. Number for zebrafish embryos used for each case is mentioned in bracket.*

**FIGURE 3:**
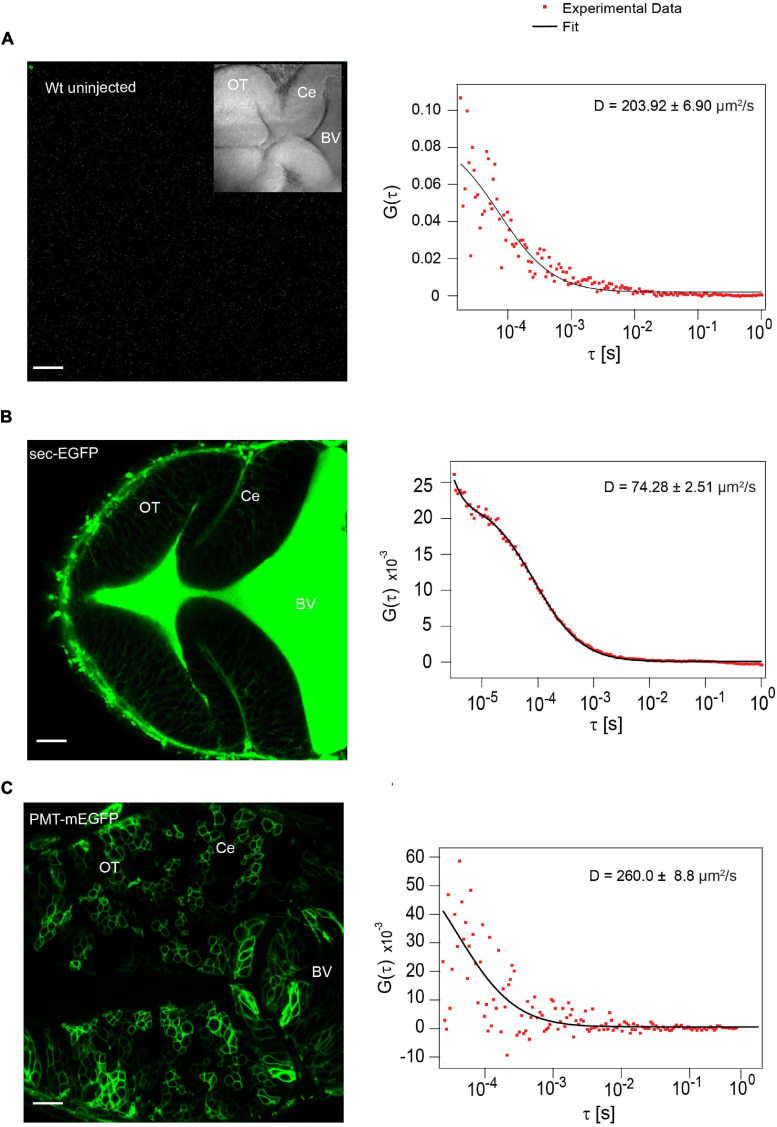
Secretion of positive and negative control in the BV **(A)** Auto fluorescence signal from wild type (Wt) unlabeled zebrafish embryo and representative autocorrelation function (ACF; dots) and fit (line) of a FCS measurement in the BV at ∼48 hpf.). **(B)** Expression of sec-EGFP in the zebrafish brain at ∼48hpf (left) and a representative autocorrelation function (ACF; dots) and fit (line) of a FCS measurement in the BV (right). **(C)** Expression of PMT-mEGFP in the zebrafish brain at ∼48 hpf (left) and a representative autocorrelation function (ACF; dots) and fit (line) of a FCS measurement in the BV of zebrafish (right). The FCS curves were fitted using 3D-2particle-1triplet model. BV, fourth brain ventricle; Ce, cerebellum; OT, optic tectum. Orientation: anterior to the left. Scale bar 20 μm.

The low intensity and high D value in the BV of unlabeled WT embryos indicates diffusion of auto-fluorescent molecules in the BV (Figure 3). When we injected embryos with the secretory peptide of Fibroblast growth factor 8a (Fgf8a) tagged with EGFP (secEGFP) as a positive control, a comparatively high molecular brightness (∼2000-2200 counts per particle per second (cps)) was observed in the BV compared to unlabeled WT embryos, and FCS analysis yielded a D of 72.90 ± 9.4 μm^2^/s, consistent with the free diffusion coefficient of EGFP (Figure 3). As a negative control, when we injected a plasma membrane targeting peptide tagged with mEGFP (PMT-mEGFP), strong signal was detected on the cell membrane whereas the molecular brightness was low (∼700–800 cps. The ACF in the BV of embryos expressing PMT-mEGFP yielded a D of 201.30 ± 42.10 μm^2^/s; similar to what was observed in the unlabeled WT embryos (Figure 3).

### C80A and S212A Mutants of Wnt3 Are Associated With Ordered Plasma Membrane Domains

**FIGURE 4:**
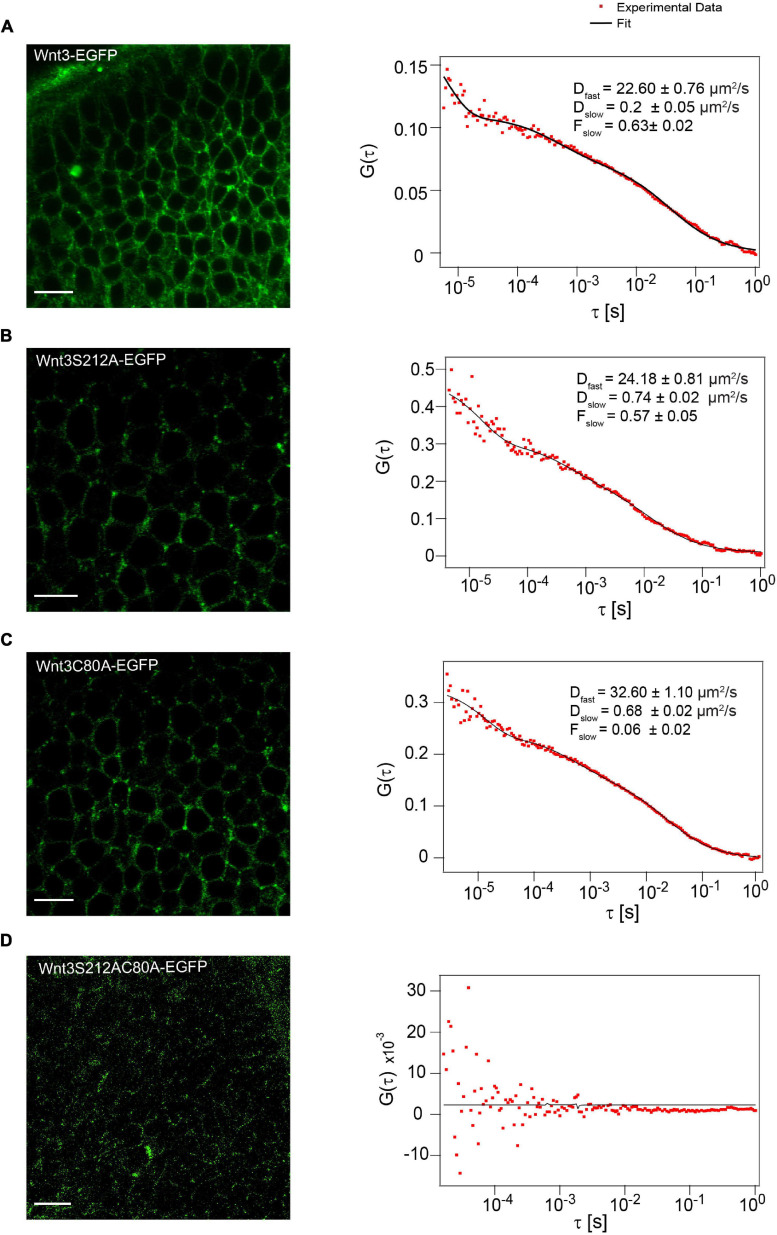
Influence of C80 and S212 lipidation on membrane localization *in vivo*. **(A)** Expression of Wnt3-EGFP on the cell membrane (left) and a representative autocorrelation function (ACF; dots) and fit (line) of a Wnt3-EGFP FCS measurement at a cell membrane (right). **(B)** Expression of Wnt3S212A-EGFP on the cell membrane (left) and a representative autocorrelation function (ACF; dots) and fit (line) of a Wnt3S212A-EGFP FCS measurement at a cell boundary (right). **(C)** Expression of Wnt3C80-EGFP on the cell membrane (left) and a representative autocorrelation function (ACF; dots) and fit (line) of a Wnt3C80A-EGFP FCS measurement at a cell membrane (right). **(D)** Expression of Wnt3S212AC80-EGFP on the cell membrane (left) (Image brightness was increased for clear visualization) and a representative autocorrelation function (ACF; dots) and fit (line) of a Wnt3S212AC80A-EGFP FCS measurement at a cell membrane (right). The FCS curves were fitted using 2D-2particle-1triplet model. BV, fourth brain ventricle; Ce, cerebellum; OT, optic tectum. Orientation: anterior to the left. Scale bar 10 μm.

Next, we investigated if C80A and S212A mutations alter the membrane association of Wnt3 in vivo. The addition of lipid groups to Wnts improves their affinity to the cell membrane and helps in their partitioning to ordered membrane domains (Gao et al., 2011; Nile and Hannoush, 2016; Azbazdar et al., 2019). This prompted us to characterize the influence of lipidation on the membrane dynamics and organization of Wnt3 *in vivo*. We first measured the dynamics of Wnt3-EGFP, Wnt3C80A-EGFP, Wnt3S212A-EGFP and Wnt3C80AS212A-EGFP in cell membranes of cells within the zebrafish brain using confocal FCS (Figure 4). As previously published, confocal FCS measurements of Wnt3-EGFP result in two fractions, a fast one (D_fast_ of 31.1 ± 8.5 μm^2^/s), representing non-membrane bound proteins either in the cytosol or extracellular space, and a slow fraction (D_slow_ of 0.6 ± 0.2 μm^2^/s; F_slow_ = 0.6 ± 0.05) that is characteristic for membrane diffusion (Veerapathiran et al., 2020). Therefore, we performed FCS on the cell membrane in the cerebellum to determine whether the mutants localize on the cell membrane similar to Wnt3-EGFP.

**FIGURE 5:**
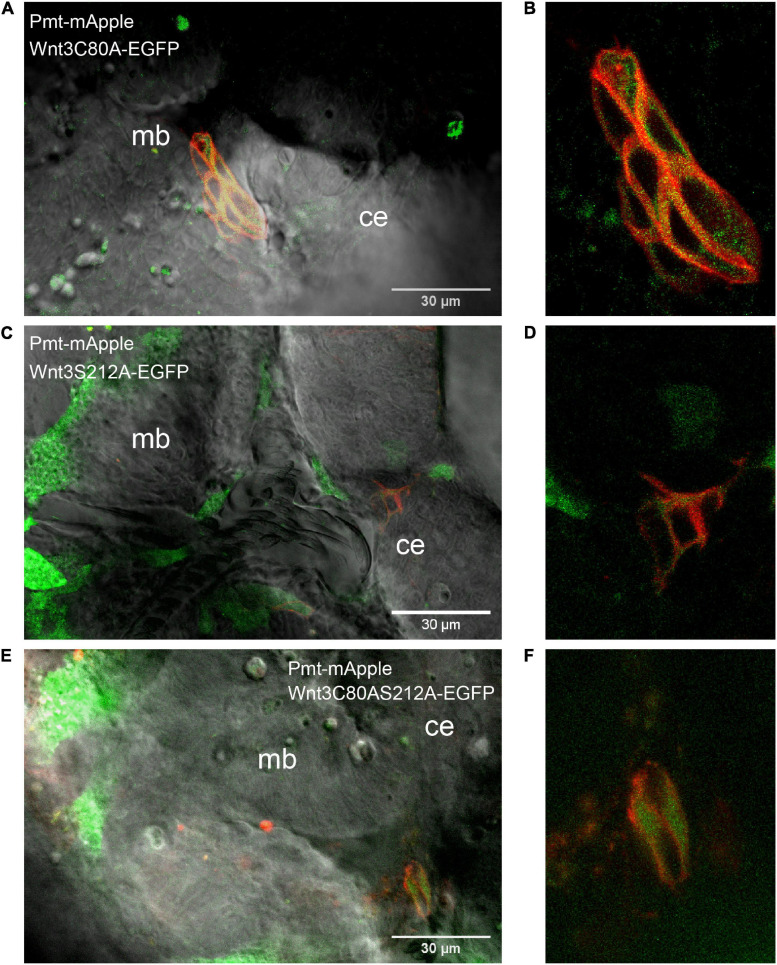
Expression profile of the mutants co expressed with PMT-mApple. Co-expression of mutants (Wnt3C80A-EGFP, Wnt3S212A-EGFP, and Wnt3S212AC80A-EGFP) and PMT-mApple in the zebrafish brain at ∼48 hpf. PMT-mApple is a protein located on the inner leaflet of the cell membrane. It is expressed under a 4kb Wnt3 promoter and serves to mark the source cells of Wnt3 expression. **(A)** Expression profile of Wnt3C80A-EGFP co-expressed with PMT-mApple in zebrafish brain. **(B)** Expression of Wnt3C80A-EGFP co-expressed with PMT-mApple in the midbrain. **(C)** Expression profile of Wnt3S212A-EGFP co-expressed with PMT-mApple in zebrafish brain. **(D)** Expression of Wnt3S212A-EGFP co-expressed with PMT-mApple in the midbrain. **(E)** Expression profile of Wnt3C80AS212A-EGFP co-expressed with PMT-mApple in zebrafish brain. **(F)** Expression of Wnt3C80AS212A-EGFP co-expressed with PMT-mApple in the midbrain.

Wnt3C80A-EGFP and Wnt3S212A-EGFP mutant constructs were both found on the cell membrane similar to Wnt3-EGFP. The correlation functions for the mutant constructs were fit with a 2D-2particle-1triplet model (see Materials and Methods), and the D for both components were computed (see Table 1). We observed a fast diffusing component (D_fast_ of 27.2 ± 7.8 μm^2^/s for Wnt3C80A-EGFP; 28.1 ± 4.9 μm^2^/s for Wnt3S212A-EGFP) and a slow diffusing component (D_slow_ = 0.6 ± 0.2 μm^2^/s and F_slow_ = 0.6 ± 0.04 for Wnt3C80A-EGFP; D_slow_ = 0.7 ± 0.3 μm^2^/s and F_slow_ = 0.5 ± 0.06 for Wnt3S212A-EGFP) (Table 1). However, Wnt3C80AS212A-EGFP is not found on the plasma membrane and we did not obtain any ACFs on the cell membrane (Figure 4). In order to locate expression of the mutants in the source and target cells, we co expressed the mutant constructs with PMT-mApple, a protein tethered to the inner leaflet of the cell membrane, which is expressed under a 4 kb Wnt3 promoter, marking source cells of Wnt3 expression. While PMT-mApple localizes to the cell membrane, Wnt3C80AS212A-EGFP is cytosolic. In case of Wnt3C80A-EGFP and Wnt3S212A-EGFP, the mutants at least partially co-localize with PMT-mApple on the cell membrane of source cells but were also found away from the source region (Figure 5).

**FIGURE 6:**
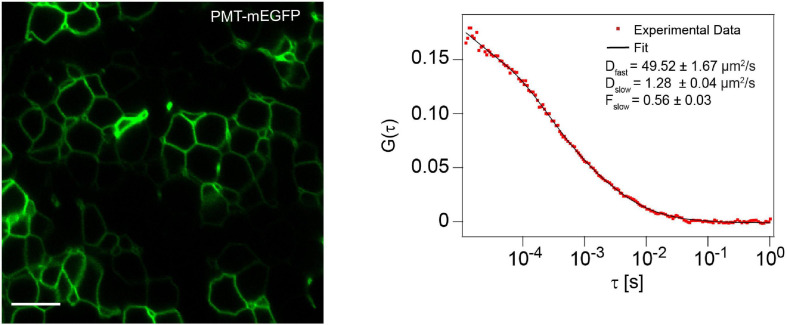
Membrane localization and dynamics of PMT-mEGFP Cell membrane localization of PMT-mEGFP (left) and a representative autocorrelation function (ACF; dots) and fit (line) of FCS measurement at the cell membrane (right). The FCS curves were fitted using 2D-2particle-1triplet model. Scale bar 10 μm.

While we measured the mobility of the membrane marker PMT-mEGFP in the cell membrane of the zebrafish brain as a control, we obtained a D_fast_ of 41.8 ± 5.0 μm^2^/s and a D_slow_ of 1.30 ± 0.40 μm^2^/s, although here the D_fast_ corresponds solely to the cytosolic fraction, as PMT-mEGFP is not secreted extracellularly (Figure 6). Overall, as the D_slow_ for the two mutants are in the same order of magnitude as Wnt3-EGFP and the membrane marker, we can infer that both Wnt3C80A-EGFP and Wnt3S212A-EGFP are localized on the membrane in zebrafish embryos. The difference in fluorescence intensities for Wnt3C80-EGFP and Wnt3-S212A-EGFP as compared to Wnt3-EGFP is due to different expression levels as the mutant plasmid constructs were injected in zebrafish embryo at 1-2 cell stage while Wnt3-EGFP is a transgenic line driven by a 4 kb Wnt3 promoter. Therefore, membrane localization for Wnt3C80-EGFP and Wnt3S212A-EGFP was confirmed based on the slow diffusion component and fraction of the slow diffusion component.

We have previously shown that Wnt3 partitions into ordered plasma membrane environments *in vivo* (Ng et al., 2016; Sezgin et al., 2017; Azbazdar et al., 2019). On establishing that Wnt3C80A-EGFP and Wnt3S212A-EGFP are localized on the membrane, we next used the SPIM-FCS diffusion law to understand the role of lipidation in its domain confinement (Figure 1). SPIM-FCS is a multiplexed FCS modality that integrates array detectors and SPIM to simultaneously quantify the in vivo dynamics of proteins at a large number of contiguous points across a large area of the sample in a single measurement (Wohland et al., 2010; Singh et al., 2013). The FCS diffusion law utilizes the scale dependence of diffusion to provide nanoscopic information on the membrane organization of the probe such as molecular trapping and meshwork compartmentalization (Lenne et al., 2006). In SPIM-FCS, this is adapted as the SPIM-FCS diffusion law by measuring the diffusion time (τ_d_) as a function of the effective observation area, A_eff_, which is adjusted by pixel binning of the image stack.

Diffusion of molecules on membranes is typically hindered through its interactions with the cytoskeletal meshwork or raft domains. As these interactions are happening at a spatial scale below the diffraction limit, an indirect way to understand the nature of hindrances is to measure the extent of deviation from free diffusion. By plotting the diffusion time (*y*-axis) vs the area a molecule transvers in space (*x*-axis) and extrapolating the plot to 0 on the *y*-axis, we obtain a *y*-intercept value (τ_0_). We can use this value to infer the type of diffusion exhibited by the molecule on the membrane. In free diffusion, as the time a molecule takes to transverse a given area scales linearly with time, the τ_0_-value is close to zero (−0.2 ≤ τ_0_ ≤ 0.2) But in cases of transient domain confinement, molecules initially take a very long time to transverse a short area, and thus the τ_0_ intercept value becomes positive (τ_0_ > 0.2). With increasing domain confinement, there is an increase in τ_0_ and conversely, reduced domain confinement leads to low τ_0_-values. A negative intercept (τ_0_ < −0.2) corresponds to cytoskeleton influenced hop-diffusion coming from initial fast dynamics within the meshwork compartments and subsequent slow down over larger areas beyond the compartment spaces (Wawrezinieck et al., 2005; Bag et al., 2016) (see section “Materials and Methods”).

**FIGURE 7:**
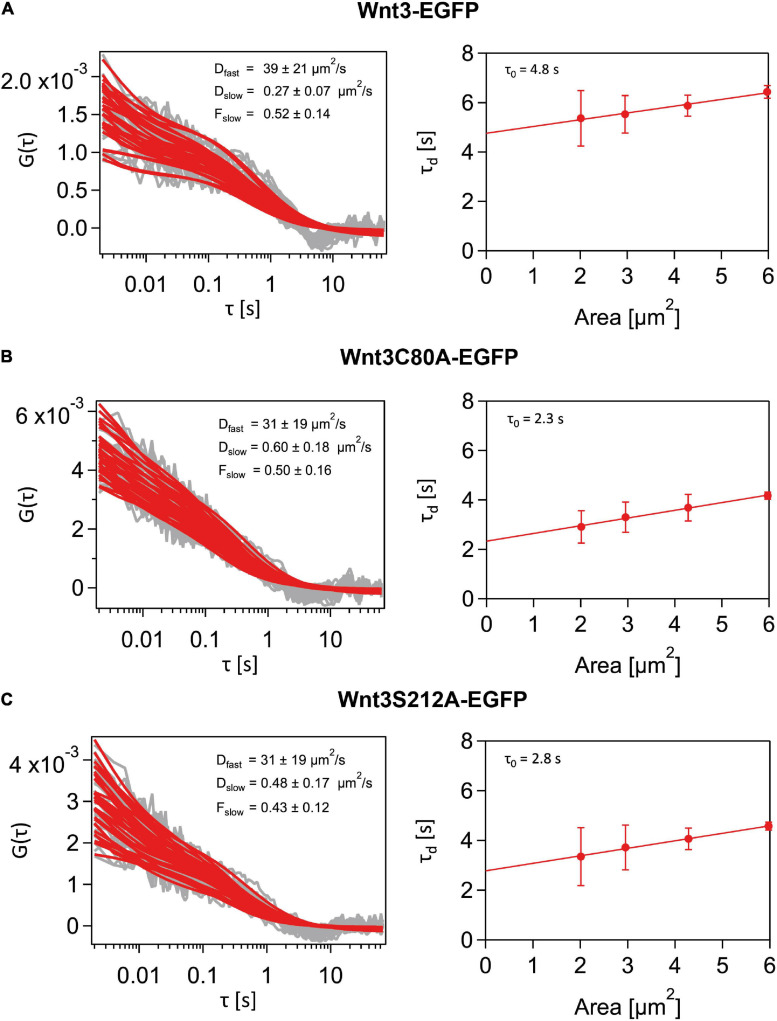
Representative SPIM-FCS and Diffusion law data from a cell. **(A)** Autocorrelation functions (left) and Diffusion law plot (right) for a Wnt3-EGFP expressing cell *in vivo.* Wnt3-EGFP expressing cells have higher τ_0_ value than the mutants. **(B)** Autocorrelation functions (left) and Diffusion law plot (right) for a Wnt3C80A-EGFP mutant-expressing cell *in vivo.* **(C)** Autocorrelation functions (left) and Diffusion law plot (right) for a Wnt3S212-EGFP mutant expressing cell *in vivo.* All autocorrelation function plots have the raw correlation data in gray and their corresponding fit in red.

As the thickness of the light sheet used is much larger than the thickness of a cell membrane, fluorescent molecules in the intracellular and extracellular spaces diffusing in 3D space also get excited. These molecules contribute to the fast component of the autocorrelation function in SPIM-FCS. The dynamics of the membrane-bound molecules diffusing on a 2D cell membrane are captured by the slow component. Thus, a two-component model, with the fast component’s fitting function using a 3D free diffusion model and the slow component’s function using a 2D free diffusion model was used to fit the ACFs. While the slow acquisition rate of the camera (∼ 1 ms) is sufficient to capture the diffusion dynamics on the membrane, it is not fast enough to accurately capture the diffusion coefficient of the fast-diffusing molecules. Nevertheless, we were able to obtain diffusion coefficient values for the fast and slow component in the same range as that of confocal FCS measurements. However, we had to set an upper bound for the diffusion coefficient of 100 μm^2^/s for the fast component to exclude erroneous fits and non-physical fit values. As we were interested in the membrane dynamics, of Wnt3 and its mutants, diffusion law analysis was performed only for the slow component. Our SPIM-FCS diffusion law analysis revealed that both Wnt3-EGFP and mutants Wnt3C80A-EGFP and Wnt3S212A-EGFP undergo domain-confined diffusion with a τ_0_ of Wnt3-EGFP (3.9 ± 1.0 s) higher than those of the mutants Wnt3C80A-EGFP (2.5 ± 0.6 s) and Wnt3S212A-EGFP (3.1 ± 0.7 s). Lower **τ_0_**-values for the mutants imply that they are less confined to ordered membrane domains as compared to Wnt3-EGFP (Table 2 and Figure 7).

**TABLE 2:** SPIM-FCS and SPIM diffusion law results for Wnt3-EGFP, Wnt3C80A-EGFP and Wnt3S212A-EGFP.

Sample	D_Fast_ [μm^2^/s] (N_ACFs_)	D_Slow_ [μm^2^/s] (N_ACFs_)	F_Slow_	τ _0_ [s]	Cells (embryos)
Wnt3-EGFP	29 ± 22 (515)	0.40 ± 0.17 (635)	0.5 ± 0.2	3.9 ± 1.0	18 (4)
Wnt3C80A-EGFP	22 ± 18 (317)	0.55 ± 0.27 (374)	0.5 ± 0.2	2.5 ± 0.6	11 (4)
Wnt3S212A-EGFP	27 ± 18 (478)	0.48 ± 0.20 (561)	0.5 ± 0.2	3.1 ± 0.7	16 (4)

*Data are mean ± S.D.*

### C80 but Not S212 Residue Is Dispensable for the Interaction of Wnt3 With Fzd1 *in vivo*

**FIGURE 8:**
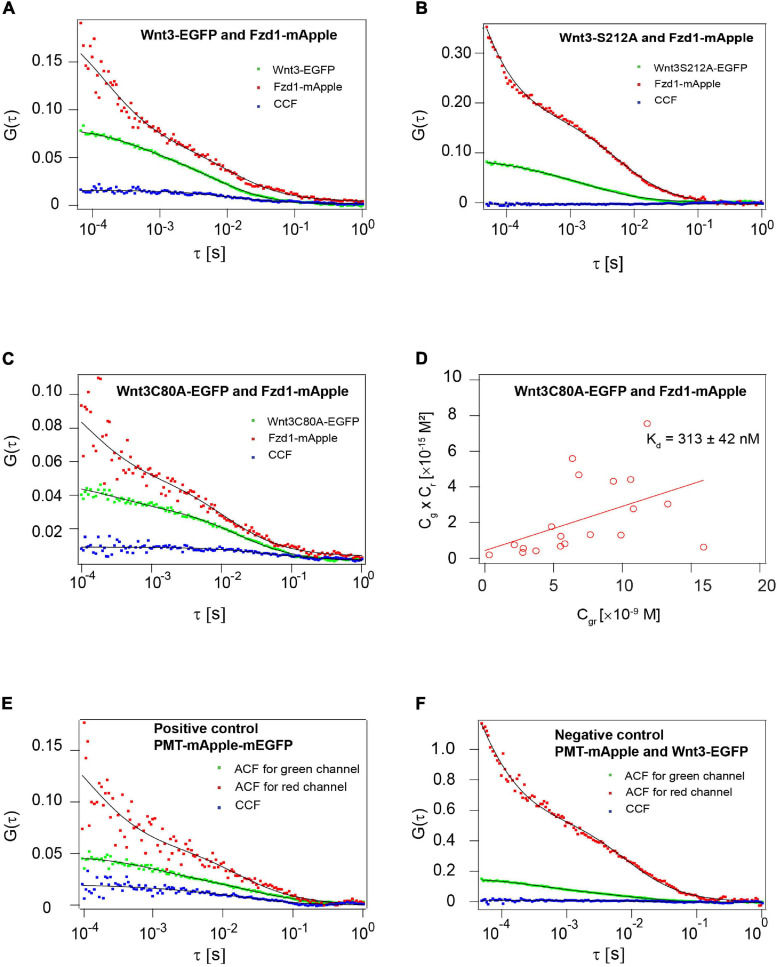
Influence of C80 and S212 lipidation on the interaction of Wnt3 with Fzd1 receptor. **(A)** Representative auto- and cross-correlation functions (dots) and fits (lines) of a Wnt3-EGFP and Fzd1mApple FCCS measurement. The positive cross-correlation function indicates Wnt3EGFP interacts with Fzd1mApple *in vivo*. **(B)** Representative auto- and cross-correlation functions (dots) and fits (lines) of a Wnt3S212A-EGFP and Fzd1mApple FCCS measurement. No cross-correlation function indicates Wnt3S212A-EGFP does not interact with Fzd1mApple *in vivo*. **(C)** Representative auto- and cross-correlation functions (dots) and fits (lines) of Wnt3C80A-EGFP and Fzd1mApple. The positive cross-correlation function indicates Wnt3C80-EGFP interacts with Fzd1mApple *in vivo*. **(D)** Determination of apparent dissociation constant (K_d_) for Wnt3C80A-EGFP and Fzd1mApple interaction *in vivo*. Cg, Cr, and Cgr represents the concentration of unbound Wnt3C80A-EGFP, unbound Fzd1mApple, and bound Wnt3C80A-EGFP and Fzd1mApple molecules, respectively. The estimated apparent K_d_ (*K*_d_ = *C*_*g*_×*C*_*r*_/*C*_*r*_*C*_*gr*_*C*_*gr*_) for Wnt3C80A-EGFP and Fzd1mApple in vivo is 313 ± 42 nM. **(E)** Representative auto- and cross-correlation functions for PMT-mApple-mEGFP which is a positive control showing a clear cross-correlation. **(F)** Representative auto- and cross-correlation functions for embryos co-expressing PMT-mApple and PMT-mEGFP as a negative control.

The high-resolution crystal structures of Wnt-Fzd complexes revealed the presence of a deep hydrophobic pocket in the CRD of Fzd in which Wnts insert their lipid tail indicating a critical role of lipidation in receptor interactions (Janda et al., 2012; Hirai et al., 2019). As we had earlier established that Wnt3 strongly interacts with Fzd1 in the dorsal cerebellum and midbrain hindbrain boundary of live zebrafish embryos with an apparent dissociation constant (K_d_) of ∼ 115 nM (Veerapathiran et al., 2020), we aimed to monitor the interactions of Wnt3C80A-EGFP and Wnt3S212A-EGFP with Fzd1-mApple in the same regions. We used quasi-PIE FCCS, a modality of FCCS that uses a pulsed laser line along with a continuous wave laser line to illuminate the sample. This approach facilitates the statistical filtering of background, spectral bleed through and detector after-pulsing (Padilla-Parra et al., 2011; Yavas et al., 2016). Embryos co-expressing Wnt3C80A-EGFP Fzd1-mApple yielded positive cross-correlations suggesting that the Wnt3C80A-EGFP retains its interaction with Fzd1 receptor in vivo. The apparent dissociation constant (K_d_) was calculated to be 313 ± 42 nM (Figure 8), signifying that C80A mutant binds with a lower affinity to Fzd1 receptor as compared to Wnt3-EGFP. Interestingly, mutation at the S212 residue abolished the Wnt3-Fzd1 interaction as no cross-correlations were observed in embryos co-expressing Wnt3S212A-EGFP and Fzd1-mApple (Figure 8). As positive control, we injected PMT-mApple-mEGFP at the one-cell stage which showed a higher cross-correlation amplitude compared to the sample, while as negative control, we co-expressed PMT-mApple and PMT-mEGFP which showed no cross-correlation (Figure 8). These results clearly suggest that the lipidation at S212 residue is vital for its in vivo interaction with Fzd1.

### C80 and S212 Lipidations Are Essential for Wnt3 to Activate β-Catenin Signaling

**FIGURE 9:**
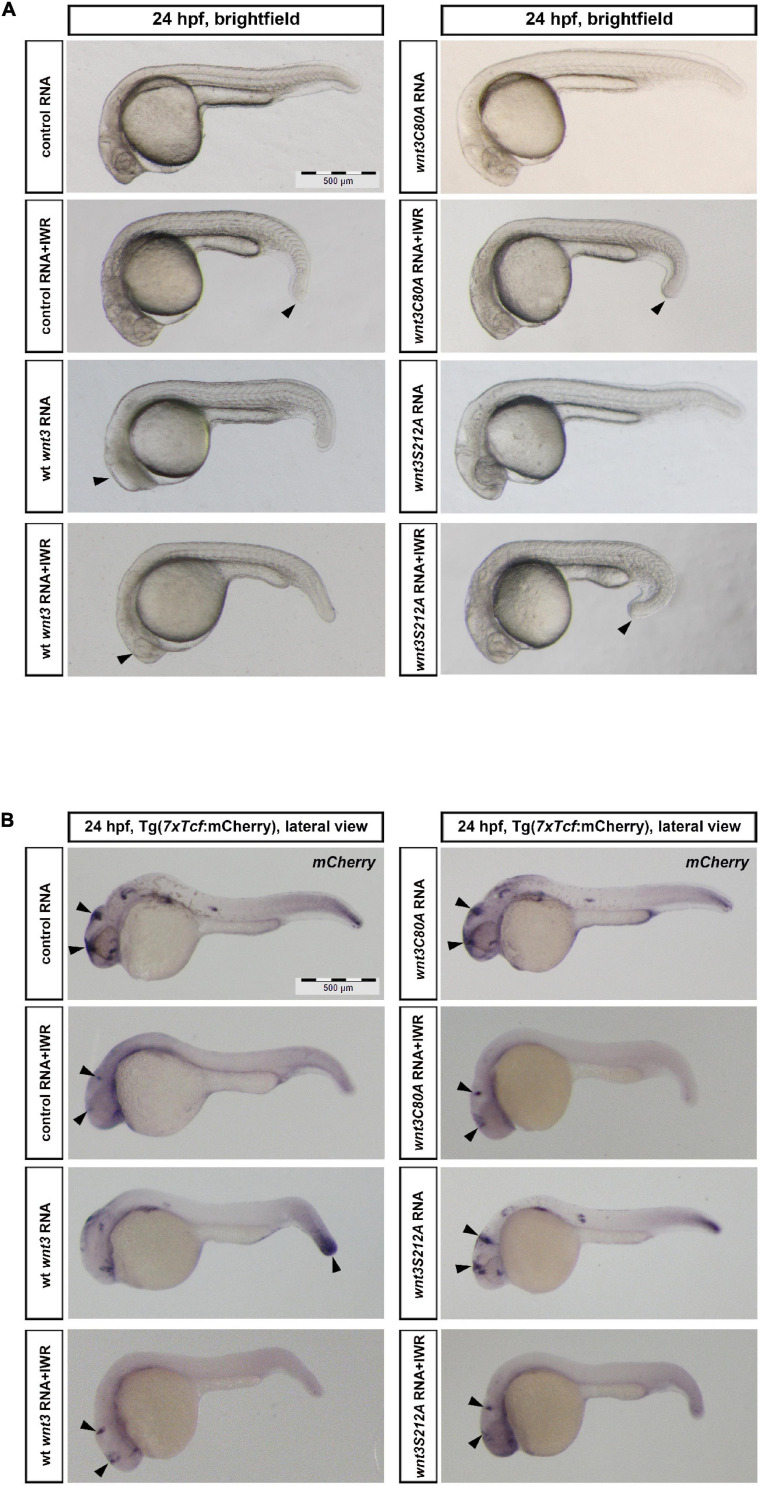
Influence of C80 and S212 residues on Wnt signaling activity **(A)** Morphological phenotypes at 24 hpf of embryos injected/treated with capped sense RNAs of control (EGFP 100 pg, 37/37 embryos), control +IWR (42/42 embryos, arrowhead: reduction in tail elongation), wt Wnt3 (100 pg, 47/53 embryos, arrowhead: loss of eye), Wnt3-EGFP + IWR (36/43 embryos, arrowhead: restoration of eye), Wnt3C80A-EGFP (100 pg, 41/43 embryos), Wnt3C80A-EGFP + IWR (41/44 embryos, arrowhead: reduction in tail elongation), Wnt3S212A-EGFP (100 pg, 52/54 embryos) or Wnt3S121A-EGFP + IWR (43/46 embryos, arrowhead: reduction in tail elongation). IWR was used at a concentration of 10 μM and DMSO was used in groups that were not treated with IWR. **(B)** Whole mount in situ hybridization (WMISH) in the transgenic Tg(*7xTcf-Xla.Siam:nlsm-Cherry^*ia*^*) canonical Wnt/ß-catenin reporter embryos showing the effects of control (EGFP 100 pg, 33/33 embryos, arrowheads: anterior Wnt expression domains), control + IWR (45/45 embryos, arrowheads: reduction in anterior Wnt expression domains), Wnt3-EGFP (100 pg, 41/49 embryos, arrowheads: increase in posterior Wnt expression domain), Wnt3-EGFP + IWR (40/45 embryos, arrowheads: restoration of anterior Wnt expression domain), Wnt3C80A-EGFP (100 pg, 45/48 embryos, arrowheads: no effect on anterior Wnt expression domains), Wnt3C80A-EGFP + IWR (38/39 embryos, arrowheads: reduction in anterior Wnt expression domains), Wnt3S212A-EGFP (100 pg, 44/46 embryos, arrowheads: no effect on anterior Wnt expression domains) or Wnt3S121A-EGFP + IWR (50/53 embryos, arrowheads: reduction in anterior Wnt expression domains) on canonical Wnt signaling. IWR was used at a concentration of 10 μM and DMSO was used in groups that were not treated with IWR1. *mCherry* WMISH shows upregulation of signaling in Wnt/ß-catenin reporter embryos by Wnt3-EGFP but not by Wnt3C80A-EGFP or Wnt3S212A-EGFP.

Lastly, to test whether lipidation of Wnt3 at the conserved cysteine and serine affects Wnt/β-catenin signaling activity, we overexpressed Wnt3-EGFP, Wnt3C80A-EGFP or Wnt3S212A-EGFP in zebrafish embryos by injecting their capped sense RNAs at the one-cell stage. We next treated the embryos with the Wnt antagonist IWR-1 for 16 h until 24 hpf. IWR treatment caused a reduction in tail elongation while Wnt3-EGFP effectively suppressed eye formation at 24 hpf, a distinctive phenotype caused by enhanced Wnt/ß-catenin signaling (Özhan et al., 2013). IWR could significantly restore the eye phenotype caused by Wnt3-EGFP. Neither Wnt3C80A-EGFP nor Wnt3S212A-EGFP overexpression exhibited any phenotypic alteration as compared to the control (Figure 9).

To examine the direct influence of the Wnt3EGFP and Wnt3 lipidation site mutant constructs on Wnt/β-catenin signaling, we took advantage of a transgenic zebrafish reporter of Wnt/ß-catenin signaling *Tg(7xTcf-Xla.Siam:nlsm-Cherry^*ia*^*) (Moro et al., 2012). IWR robustly inhibited reporter activity in all domains of expression. In contrast to strong reporter activation by Wnt3-EGFP in the posterior site of embryos at 24 hpf, Wnt3C80A-EGFP or Wnt3S212A-EGFP overexpression had no detectable effect on the reporter activity as shown by whole mount in situ hybridization (WMISH) (Figure 9).

**FIGURE 10:**
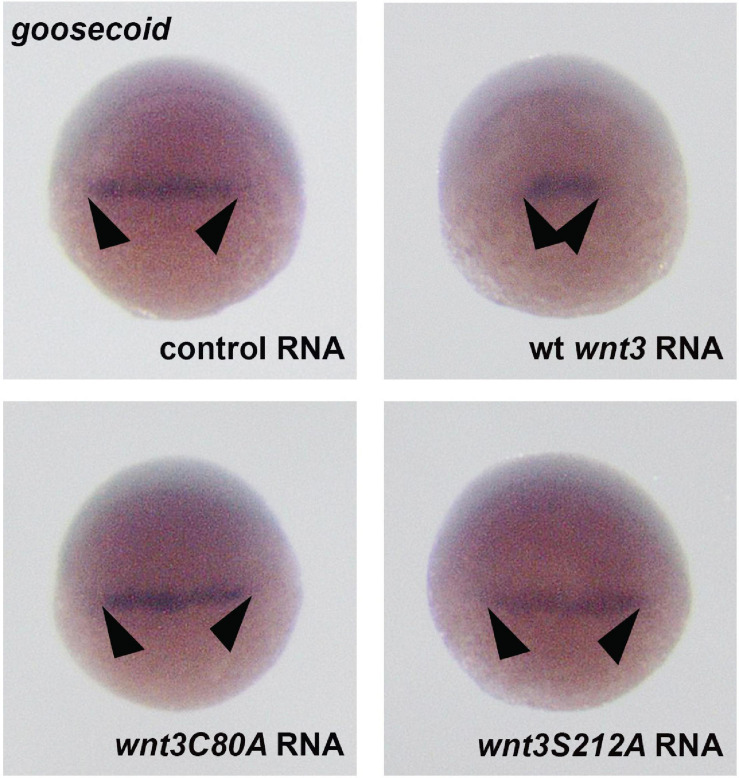
Whole mount *in situ* hybridization for the dorsal organizer marker gene *goosecoid* (*gsc*) in wt embryos at the shield stage, showing the effects of control (EGFP 100 pg, 35/35 embryos, arrowheads: normal *gsc* expression domain), Wnt3-EGFP (100 pg, 31/35 embryos, arrowheads: reduction in *gsc* expression domain), Wnt3C80A EGFP (100 pg, 38/41 embryos, arrowheads: no effect on *gsc* expression domain), and Wnt3S212A-EGFP (100 pg, 32/34 embryos, arrowheads: no effect on *gsc* expression domain).

During gastrulation, canonical β-catenin signaling mediates specification of the ventrolateral mesoderm by repressing the dorsal organizer (Lekven et al., 2001). To test whether Wnt3 lipidation affects mesodermal patterning of the embryo, we injected mRNAs of Wnt3 constructs into 1-cell embryos and examined development of the organizer at early development. While Wt Wnt3 caused a significant restriction of the dorsal organizer region marked by *goosecoid* (*gsc*) expression (Yao and Kessler, 2001) at early gastrula (shield, 6 hpf) stage, neither Wnt3C80A-EGFP nor Wnt3S212A-EGFP altered the size of organizer region as compared to control (Figure 10). Taken together, these data indicate that lipid modifications of Wnt3 at both C80 and S212 are necessary for downstream activation of Wnt/β-catenin signaling.

These results show that point mutations at either S212 or C80 residue of Wnt3 do not impede its membrane localization and secretion, however, localization to the ordered membrane domains is compromised. There are two possible explanations for this: (i) Wnt3 is dually lipidated at its C80 and S212 position, and the lipid tail at one residue compensates for the absence of the lipid moiety at the mutated residue (ii) Wnt3 is lipidated only at S212, but its membrane localization and secretion is independent of its lipidated.

## Discussion

Post-translational lipidation involves the covalent modification of proteins with lipids in the cytoplasm or in the lumen of the organelles involved in the secretory pathway. Among the secreted signaling molecules regulating development, Wnts and Hedgehog are the most commonly reported lipid modified morphogens with diverse functions (Parchure et al., 2018). Almost all Wnts, except WntD in *Drosophila*, are post-translationally lipidated, and this lipid modification has a regulatory role in mediating membrane-protein interaction, secretion and signaling activity (Nadolski and Linder, 2007; Jiang et al., 2018). Initial mass spectrometry studies marked cysteine and serine as putative lipid addition sites (Willert et al., 2003; Takada et al., 2006). Later however, the crystal structure of XWnt8 (Janda et al., 2012), along with biochemical studies using metabolic labeling and click chemistry (Gao and Hannoush, 2014; Miranda et al., 2014), identified serine as the solitary lipidation site across all Wnts. This was further supported by functional studies where the Wnt3a S209A mutant expressed in L-cells was not secreted into the media (Takada et al., 2006) and the S239A Wg mutant diminished downstream Wnt signaling in Drosophila imaginal disks (Franch-Marro et al., 2008). However, in a few Wnt proteins, mutating the conserved serine residue did not perturb their in vivo expression, secretion, or function, which cannot be explained by a single lipid addition site. Additionally, the conformation of Wnts could also differ in its free and bound state (Janda and Garcia, 2015). Therefore, in this work, we investigated the possibility of lipid modifications at the conserved serine and cysteine residues and the importance of lipidation in Wnt trafficking and activity.

The addition of hydrophobic groups to soluble proteins increases their affinity to membranes, thereby influencing their membrane organization and function. Furthermore, these lipid modifications also drive their compartmentalization in organized membrane domains which serve as portals for protein-protein interactions, oligomerization, and signaling among others (Levental et al., 2010a,b; Lingwood and Simons, 2010; Resh, 2013). As zebrafish Wnt3 is known to partition into cholesterol rich membrane domains and inhibition of Porc by C59 is known to decrease its association with these domains, here we monitored the membrane localization and domain compartmentalization of C80A and S212A Wnt3EGFP mutants in the zebrafish brain. From our diffusion law results, we observed that both Wnt3C80A-EGFP and Wnt3S212A-EGFP mutants are found on the membrane albeit, with a lower degree of domain confinement as compared to Wnt3-EGFP embryos. However, the double mutant Wnt3C80AS212A-EGFP construct, was not detected on the membrane.

Our results demonstrate that disrupting either the conserved cysteine or conserved serine does not abolish membrane localization for Wnt3 in the zebrafish brain. However, its association with ordered domains is reduced as shown by the lower τ_0_-values for the mutants (Wnt3S212A-EGFP and Wnt3C80A-EGFP) as compared to Wnt3-EGFP. This suggests that lipidation at either residue is sufficient for membrane binding of Wnt3 in zebrafish but lipidation at both residues is required for its correct partitioning into ordered membrane domains. This can be seen by comparing τ_0_-values for the mutants (Wnt3C80A-EGFP and Wnt3S212A-EGFP) with Wnt3-EGFP, which are lower and thus indicate reduced confinement to ordered membrane domains.

The BV serves as a route for signaling molecules to achieve long-range transport in the central nervous system (Kaiser et al., 2019). In the zebrafish brain, Wnt3 is secreted into the BV as it is adjacent to the Wnt3-expressing cells of the cerebellar rhombic lip. So next, we studied how mutations in Wnt3 C80 and S212 residues affect their secretion into the BV. Similar to our results on membrane organization, we observed that Wnt3C80A-EGFP and Wnt3S212A-EGFP mutants are secreted into the BV, while mutating both residues simultaneously renders Wnt3 incapable of secretion. This further substantiates the possibility that Wnt3 in zebrafish is lipidated at both its C80 and S212 position. These results are consistent with another study in which Tang et al. (2012) observed membrane localization and secretion of single Wg C93A and S239A mutants but not of Wg double mutants.

Indeed, lipid modifications are believed to mediate the interaction of Wnt with Wls, the intracellular Wnt chaperone protein that transports Wnts to the membrane and facilitates their secretion (Bartscherer et al., 2006; Herr and Basler, 2012). Blocking the interaction of Wnt with Wls or mutating Wls leads to intracellular accumulation of Wnt proteins and degradation (Bänziger et al., 2006). As we note in this study that Wnt3C80A-EGFP and Wnt3S212A-EGFP single mutants are targeted to the membrane and secreted into the BV, while Wnt3C80AS212A-EGFP is not, we can assume that a single lipid modification in the Wnt3 single mutants maintains their interaction with Wls, while the lack of lipidation at both residues diminishes Wnt3-Wls interaction. However, how mutations at C80 and S212 residue modulate the interaction of Wnt3 with Wls *in vivo* requires further investigation.

Overall, disruption of Wnt lipidation at C80 or S212 does not abolish membrane binding as shown by the slow diffusion coefficient for Wnt3C80A-EGFP and Wnt3S212A-EGFP mutant which is of the same order as the slow diffusion coefficient for Wnt3-EGFP. However, neither mutant can activate downstream signaling. This lack of downstream signaling might be a result of reduced localization of mutants to the ordered lipid membrane domains which is demonstrated by the lower τ_0_ values for the mutants as compared to Wnt3-EGFP. The importance of membrane organization of proteins has been discussed by Azbazdar et al. (2019) who have proposed that Wnt3 acylation is necessary for its localization to ordered domains in the membrane and subsequently to downstream signaling. It is possible that partitioning into the ordered membrane domains helps in Wnt complex signalosome formation which leads to the activation of the signaling pathway. However, this needs further investigation.

Finally, when secreted Wnt3 reaches its target tissues, how do covalent lipid modifications of Wnt3 regulate receptor binding and signaling activity? To answer this question, we measured how the mutations at C80 and S212 residue impact Wnt3 interaction with Fzd1 using quasi-PIE FCCS. We found that Wnt3C80A-EGFP maintains its interaction with Fzd1-mApple, albeit with an almost three times lower binding affinity when compared to Wnt3-EGFP. In contrast, Wnt3-EGFP is unable to interact with Fzd1-mApple *in vivo* when the S212 residue is substituted with alanine. Our results are in line with the description of the serine residue positioning its lipid adduct into the hydrophobic pocket of Fzd as determined from the crystal structure of xWnt8-mFzd8 complex. Interestingly, we observed that despite Wnt3C80A-EGFP binding to Fzd1 receptor, it does not activate downstream signaling. Although Azbazdar et al. also reported that non-acylated Wnt3 interacts with Fzd8 but fails to activate downstream β-catenin signaling in HEK293 cells (Azbazdar et al., 2019), the reason still remains elusive. However, note that these observations are tissue-specific and context dependent and results might vary if the same Wnt is being studied in a different tissue/region in zebrafish or in a different organism. Finally, we analyzed the effect of C80A and S212A mutation on the downstream signaling pathway and found that Wnt3C80A-EGFP and Wnt3S212A-EGFP failed to activate the β-catenin signaling pathway as demonstrated by whole mount in situ hybridization for the *goosecoid* (*gsc*)

Collectively, the *in vivo* functional studies in this work indicate that zebrafish Wnt3 experiences lipid modifications at its conserved cysteine and serine residues, which regulate its secretion and activity in neural cells. However, additional investigations are required to further substantiate our results. First, a detailed mass-spectrometric characterization of Wnt3 purified from zebrafish neural cells is required to precisely identify the type of lipids and the sites of lipidation in zebrafish Wnt3. Moreover, palmitoylation, the most commonly reported form of lipid modification in Wnts, has a unique characteristic of being reversible (Ganesan and Levental, 2015; Azbazdar et al., 2019). Hence, it is critical to check whether Wnt3 undergoes a series of palmitoylation and depalmitoylation events and how that affects its distribution and function *in vivo*. Lastly, the signaling range of Wnts in vivo could also be lipidation dependent, as shown by Baena-Lopez et al. (2009) for Wg in *Drosophila*. Therefore, it is also essential to examine how changes in lipidation modulates the range and gradient kinetics of Wnt3 during vertebrate brain development.

In conclusion, we infer that zebrafish Wnt3 is dually lipidated at its C80 and S212 residue. We find that single mutants at C80 or S212 are both binding to membranes, albeit with reduced domain localization, and are secreted. But only Wnt3C80A-EGFP binds to its receptor Fzd1, however without activating signaling in zebrafish neural cells. The double mutant Wnt3C80AS212A-EGFP is neither found on the membrane nor is it secreted. Overall, this study sets the framework for a detailed understanding of the molecular basis of Wnt lipidation during mammalian brain development and developing novel drugs for diseases that therapeutically target the Wnt signaling network.

## Data Availability Statement

The original contributions presented in the study are included in the article/supplementary material, further inquiries can be directed to the corresponding author/s.

## Ethics Statement

The animal study was reviewed and approved by NUS Institutional Animal Care and Use Committee (IACUC) breeding protocol BR18-1023.

## Author Contributions

TW, SV, and GO designed the experiments. SV and DD performed the FCS and FCCS experiments. AN performed and analyzed the SPIM-FCS measurements. YA performed the phenotype characterization and reporter line assay for downstream Wnt β-catenin signaling. CT prepared the constructs and transgenic zebrafish lines. SV, DD, GO, and TW wrote the manuscript. All authors contributed to the discussion. All authors contributed to the article and approved the submitted version.

## Conflict of Interest

The authors declare that the research was conducted in the absence of any commercial or financial relationships that could be construed as a potential conflict of interest.

## Footnotes

1 https://www.dbs.nus.edu.sg/lab/BFL/index.html

2 https://github.com/ImagingFCS/Imaging_FCS_1_52
